# The RhoGEF Trio Functions in Sculpting Class Specific Dendrite Morphogenesis in *Drosophila* Sensory Neurons

**DOI:** 10.1371/journal.pone.0033634

**Published:** 2012-03-19

**Authors:** Srividya Chandramouli Iyer, Dennis Wang, Eswar Prasad R. Iyer, Sarah A. Trunnell, Ramakrishna Meduri, Riaz Shinwari, Mikolaj J. Sulkowski, Daniel N. Cox

**Affiliations:** 1 School of Systems Biology, George Mason University, Manassas, Virginia, United States of America; 2 Krasnow Institute for Advanced Study, George Mason University, Fairfax, Virginia, United States of America; VIB and KU Leuven, Belgium

## Abstract

**Background:**

As the primary sites of synaptic or sensory input in the nervous system, dendrites play an essential role in processing neuronal and sensory information. Moreover, the specification of class specific dendrite arborization is critically important in establishing neural connectivity and the formation of functional networks. Cytoskeletal modulation provides a key mechanism for establishing, as well as reorganizing, dendritic morphology among distinct neuronal subtypes. While previous studies have established differential roles for the small GTPases Rac and Rho in mediating dendrite morphogenesis, little is known regarding the direct regulators of these genes in mediating distinct dendritic architectures.

**Methodology/Principal Findings:**

Here we demonstrate that the RhoGEF Trio is required for the specification of class specific dendritic morphology in dendritic arborization (da) sensory neurons of the *Drosophila* peripheral nervous system (PNS). Trio is expressed in all da neuron subclasses and loss-of-function analyses indicate that Trio functions cell-autonomously in promoting dendritic branching, field coverage, and refining dendritic outgrowth in various da neuron subtypes. Moreover, overexpression studies demonstrate that Trio acts to promote higher order dendritic branching, including the formation of dendritic filopodia, through Trio GEF1-dependent interactions with Rac1, whereas Trio GEF-2-dependent interactions with Rho1 serve to restrict dendritic extension and higher order branching in da neurons. Finally, we show that *de novo* dendritic branching, induced by the homeodomain transcription factor Cut, requires Trio activity suggesting these molecules may act in a pathway to mediate dendrite morphogenesis.

**Conclusions/Significance:**

Collectively, our analyses implicate Trio as an important regulator of class specific da neuron dendrite morphogenesis via interactions with Rac1 and Rho1 and indicate that Trio is required as downstream effector in Cut-mediated regulation of dendrite branching and filopodia formation.

## Introduction

The elaboration of class specific dendritic architectures is a hallmark of neuronal subtype as well as a key determinant in neuronal connectivity and the formation of functional neural networks. Studies to date, in both vertebrates and invertebrates, have demonstrated that the acquisition of class-specific dendrite morphologies is subject to regulation by complex genetic and molecular programs involving both intrinsic factors and extrinsic cues [1]–[3]. *Drosophila* dendritic arborization (da) sensory neurons have proven a powerful model system in which to investigate the molecular mechanisms governing class specific dendritic architecture and receptive field specification revealing important roles for a broad range of biological processes including transcriptional regulation, cytoskeletal regulation, cell signaling, and cell-cell interactions [2], [4], [5].

As dendritic development is a highly dynamic process, modulation of the cytoskeleton provides a key mechanism by which to effect changes in morphology which can manifest in alterations in function and neuronal connectivity underlying such biologically relevant events as synaptic plasticity. Cytoskeletal regulators have been demonstrated to exert significant influence on dendrite morphogenesis by regulating both actin and microtubule organization within complex class specific arbors [6], [7]. The Rho-family of small GTPases, including Rac, Rho, and Cdc42, as well as certain downstream effectors, have been demonstrated to play a pivotal role in regulating actin dynamics during dendrite and dendritic spine morphogenesis [8]–[12] and moreover, defects in Rho GTPase signaling have been implicated in various forms of mental retardation [13]. In addition, these small GTPases exert differential effects on neuron development with activation of Rac and Cdc42 functioning to promote neurite extension, whereas RhoA/Rho1 activation mediates neurite retraction. For example, in vertebrates, studies have demonstrated that Rho GTPases are activated by sensory stimuli and that activity-dependent dendritic growth requires activation of Rac1 and Cdc42, and decreased RhoA activation [14], [15]. In *Drosophila*, RhoA/Rho1 has been implicated in restricting dendritic outgrowth in both mushroom body neurons [16] and in da neurons where is it negatively regulated by the RhoGAP Crossveinless-c [17]. In contrast, Rac1 has been demonstrated to promote dendritic branching complexity in da neurons [18]–[21] as well as LPTC neurons in the CNS where it modulates the number of dendritic spines [22]. Intriguingly, another recent study revealed molecular mechanisms by which the class specific transcription factor activity of the Knot/Collier and Cut proteins act to regulate dendrite arborization in da neurons. This study further demonstrated Cut and Knot/Collier differentially interact with Rac1 in mediating dendritic filopodia formation or branch formation, respectively [23]. Despite significant evidence for the differential roles of Rac and Rho in mediating dendrite morphogenesis, little is known regarding the direct regulators of these small GTPases in this developmental process.

A strong candidate regulator for both Rac and Rho in da neurons is the multi-functional domain protein Trio which encodes a guanine nucleotide exchange factor (GEF) involved in the activation of small GTPases. *Drosophila* Trio, together with its evolutionarily conserved orthologs in *C. elegans* and mammals, is a member of the Dbl homology (DH) family of GEF proteins. Trio contains two independent GEF domains, GEF1 and GEF2, which have been demonstrated in vertebrates to differentially activate Rac and Rho, respectively [24], [25]. Previous studies implicate *Drosophila* Trio, as well as its orthologs in *C. elegans* and mammals, in mediating axon guidance, neurite extension, and cell motility in diverse neuronal subtypes [26]–[31]. Comparatively, little is known regarding Trio function in *Drosophila* dendrite development, whereas the Trio ortholog, Kalirin-7, has been demonstrated in vertebrates to mediate dendritic spine morphogenesis and synaptic plasticity [32]–[35].

Trio has further been demonstrated to act via a GEF1-dependent activation of Rac1 in mediating photoreceptor and motor neuron axon guidance in *Drosophila* [28], [30], [36], whereas the GEF2 domain has been implicated in regulating synaptic transmission and pharynx musculature pumping in *C. elegans* [37]. Moreover, recent studies have identified Trio as a downstream effector of Liprin [38] and retrograde BMP signaling [39] in mediating synaptic growth and stabilization via Trio GEF1-dependent activity at R7 photoreceptor and neuromuscular junction synapses. In contrast to these GEF1-dependent activities mediated via Rac1, little is known regarding the putative function of the *Drosophila* Trio GEF2 domain *in vivo* with respect to potential regulation of Rho1.

Here we demonstrate that Trio is expressed in all da neuron subclasses where it plays an important functional role in sculpting class-specific dendrite morphologies. Through both loss-of-function and gain-of-function analyses, we reveal cell autonomous requirements for Trio in regulating dendritic branching complexity in da neuron subtypes. We show that Trio promotes dendritic branching via GEF1-dependent interaction with Rac1 and that Trio can restrict dendritic branching via GEF2-dependent interaction with Rho1. We further demonstrate that Cut induced *de novo* dendritic branching and filopodia formation require Trio activity suggesting these molecules may act in a pathway to regulate dendritogenesis.

## Results

### Trio is expressed in all da neuron subclasses

**Figure 1:**
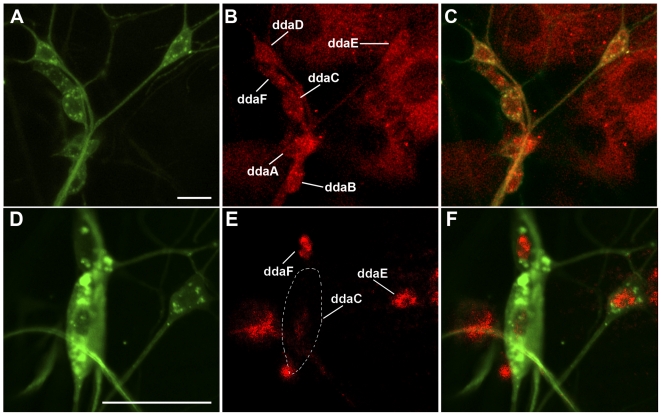
Trio is expressed in all da neuron subclasses. (**A–C**) Confocal images of wild-type third instar larval filet doubled stained with anti-HRP-488 (**A**) and anti-Trio (**B**) antibodies reveals specific localization of Trio to all dorsal cluster da neuron cell bodies. Trio is also expressed in other dorsal cluster PNS neurons labelled by anti-HRP, including external sensory (es) neurons. (**D–F**) The class IV da neuron specific *ppkGAL4,UASmCD8::GFP* reporter strain to drive expression of *UAS-trio^RNAi^* in class IV neurons followed by anti-HRP-488 (**D**) and anti-Trio labelling (**E**). These analyses revealed strong knockdown of Trio specifically in the class IV ddaC neuron (outlined), but did not disrupt Trio expression in adjacent class I (ddaE) and class III (ddaF) da neurons. Scale bars represent 30 microns.

While previous studies have demonstrated strong Trio expression in embryonic CNS neurons, photoreceptor axons, mushroom body neurons, and muscle attachment sites [27]–[30], expression in PNS neurons has not been described. To investigate Trio expression in the PNS da neurons, we doubled-labeled wild-type third instar larval filets with anti-Trio antibody and a fluorescently conjugated HRP antibody which specifically labels all PNS neurons. Analyses of the dorsal cluster of da neurons revealed Trio expression in the cell bodies of all da neuron subclasses (Fig. 1A–C). To assess Trio antibody specificity, we used the class IV da neuron specific *ppkGAL4,UASmCD8::GFP* reporter strain to drive expression of *UAS-trio^RNAi^* followed by anti-Trio labeling. As compared to wild-type Trio expression (Fig. 1B), these analyses revealed strong, specific knockdown of Trio protein expression in class IV neurons without disrupting Trio expression in other adjacent da neuron subclasses. Moreover, these analyses demonstrated that the *UAS-trio^RNAi^* transgene is capable of mediating strong loss of function knockdown for the *trio* gene product (Fig. 1D–F).

### Trio acts cell-autonomously in promoting class I da neuron dendrite branching and growth

**Figure 2:**
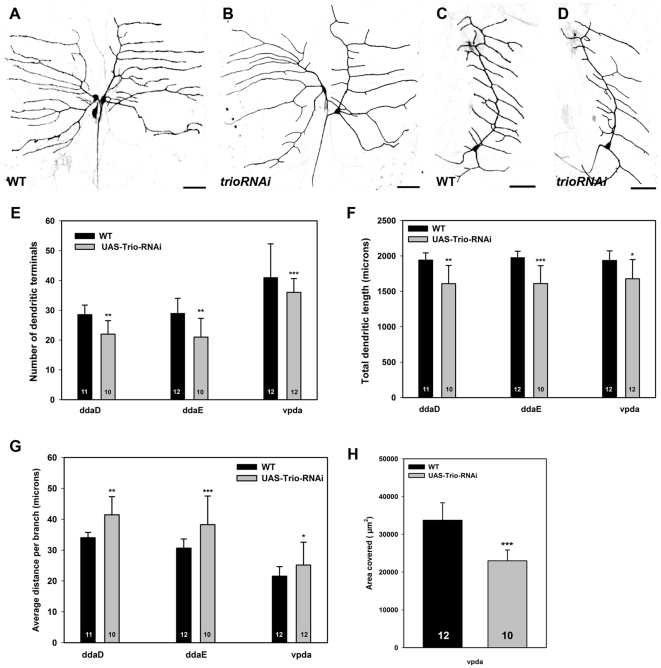
Trio promotes class I da neuron dendritic branching and field coverage. (**A–D**) Live confocal images of third instar larval dorsal (ddaD/E) and ventral (vpda) class I da neurons labeled with *GAL4^221^,UAS-mCD8::GFP*. As compared to controls (**A,C**), *UAS-trio^RNAi^* knockdown results in reduced dendritic branching in ddaD/E (**B)** and vpda (**D**) class I neurons. (**E–H**) Quantitative analyses of dendritic branching, extension, and area in *trio^RNAi^* relative to controls. (**E**) *trio* knockdown leads to a significant reduction in the average number of dendritic terminals reflecting an overall reduction in dendritic branching in all class I neurons. (**F**) Disruption of *trio* results in an overall reduction in total dendritic length in all class I neurons. (**G**) The average length per dendritic branch is increased in all class I neurons following *trio* knockdown. (**H**) Relative to control, *trio^RNAi^* knockdown reduces total dendritic area of vpda neurons. Images were taken at 20× magnification and size bar represents 50 microns. The total *n* value for each neuron and genotype quantified is reported on the bar graph. Statistically significant *p* values are reported on the graphs as follows (* = *p*<0.05; ** = *p*<0.01; *** = *p*<0.001). Genotypes: **WT**: *GAL4^221^,UAS-mCD8::GFP*/+. ***trio^RNAi^***: *GAL4^221^,UAS-mCD8::GFP/UAS-trio^RNAi^*.

To assess the potential role of *trio* in mediating class I dendritogenesis, we conducted loss of function analyses using the *trio^RNAi^* transgene. Class I specific knockdown of *UAS-trio^RNAi^* via *GAL4^221^,UAS-mCD8::GFP* expression revealed a reduction in dendritic branching in all class I da neurons (Fig. 2A–D). To assess changes in dendritic branch complexity among *trio* mutant class I neurons relative to wild-type controls, we quantitatively analyzed the average number of dendritic terminals. These analyses revealed statistically significant reductions in dendritic branching as measured by fewer dendritic terminals in all *trio* mutant class I neurons (Fig. 2E). Quantitative analyses further revealed that the reductions in dendritic branching led to overall reductions in total dendritic length in *trio* mutants as compared to controls (Fig. 2F). While the number of dendritic branches and overall dendritic length were reduced in *trio* mutants, we discovered that the average length per dendritic branch was increased relative to controls (Fig. 2G) as a result of the reduction in overall branching. Dendritic branch order analyses in vpda neurons, however, did not reveal any appreciable change in *trio* knockdown relative to control indicating that despite reductions in overall dendritic branching and length, the order among remaining branches was not significantly altered (data not shown). Finally, analyses of total dendritic area in class I vpda neurons revealed that *trio* is required to promote dendritic growth and field coverage (Fig. 2H). Collectively, these analyses demonstrate that Trio functions cell-autonomously in class I da neurons to promote dendritic branching and growth/extension.

### Trio promotes dendritic branching and filopodia formation in class III da neurons

**Figure 3:**
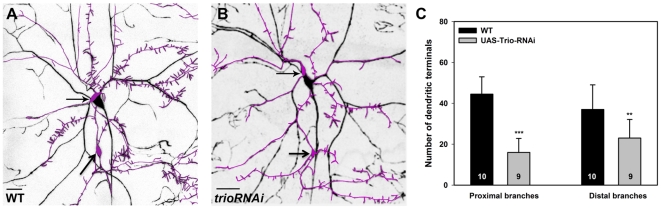
Trio is required for dendritic branching and the formation of filopodia in class III da neurons. (**A,B**) Live confocal images of third instar larval dorsal class III and IV da neurons labeled by the F-actin reporter, *UAS-GMA*, and driven by the *ppk-GAL4* transgene. Class III da neurons are distinguished by the presence of short, actin-rich dendritic filopodia emanating from the primary branches. The class III ddaA and ddaF neuron cell bodies are indicated by the arrows and for clarity the class III neuron cell bodies and dendrites have been highlighted by a magenta pseudo-color trace overlay. As compared to wild-type controls (**A**), *UAS-trio^RNAi^* knockdown results in a strong reduction in dendritic branching particularly with respect to the characteristic dendritic filopodia in class III neurons (**B**). (**C**) Quantitative analyses of the average number of dendritic terminals reveal significant reductions in branching proximal and distal to the cell body. For these analyses, 100×100 micron boxes were drawn in parallel areas proximal and distal to the cell body in both wild-type and *trio^RNAi^* and the average number of dendritic terminals quantified. Images were taken at 20× magnification and size bar represents 50 microns. The total *n* value for each neuron and genotype quantified is reported on the bar graph. Statistically significant *p* values are reported on the graph as follows (** = *p*<0.01; *** = *p*<0.001). Genotypes: **WT**: *UAS-GMA/+;ppk-GAL4*/+;+. ***trio^RNAi^***: *UAS-GMA/+;ppk-GAL4/+;UAS-trio^RNAi^/+*.

Class III da neurons are characterized by the presence of short “spine-like” dendritic filopodia which emanate from the primary branches [40]. Previous studies have demonstrated that these dendritic filopodia are actin-rich processes subject to regulation by Rac1 [19], [21], [23]. To assess the potential role of *trio* in regulating class III dendrite morphogenesis, we analyzed the effects of *trio^RNAi^* knockdown within these neurons. To achieve this we took advantage of a *ppk-GAL4* insertion on the second chromosome which marks both class III and IV da neurons which we combined with the F-actin reporter transgene *UAS-GMA* [41] allowing for the simultaneous assessment of class III morphology and distribution of actin-rich processes within the dendritic arbors. As compared to controls, knockdown of *trio* resulted in a dramatic reduction in the formation of the characteristic class III dendritic filopodia (Fig. 3A, 3B). Quantitative analyses demonstrate reductions in dendritic branch terminals both proximal and distal to the cell body (Fig. 3C). These analyses demonstrate the Trio is cell-autonomously required to promote dendritic branching and the formation of actin-rich dendritic filopodia in class III neurons.

### Trio promotes dendritic branching complexity and field coverage in class IV da neurons

**Figure 4:**
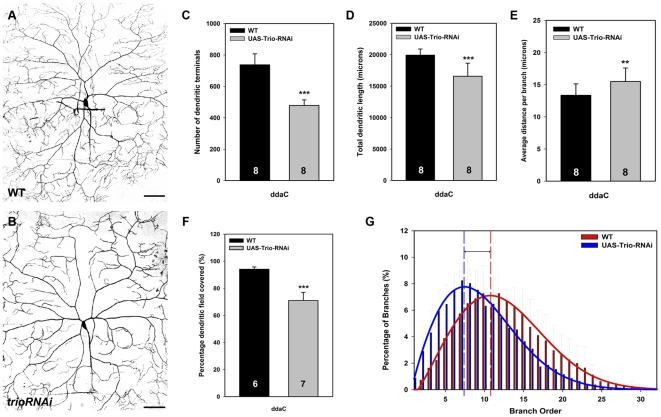
Trio regulates higher order dendritic branching and field coverage in class IV da neurons. (**A,B**) Confocal live images of the dorsal cluster class IV ddaC neuron labeled by the class IV specific reporter *GAL4^477^,UAS-GFP* at the the third instar larval stage of development. (**A**) Wild-type class IV ddaC neuron characterized by full coverage of dendritic field and highly complex dendrite branching pattern particularly at dendritic termini. (**B**) Class IV ddaC neuron expressing a *UAS-trio-RNAi* transgene. The loss-of-function *trio* phenotype is characterized by a dramatic reduction in dendrite branching complexity at both proximal primary branches and distal dendritic terminal branches. (**C–G**) Quantitative analyses of dendritic branching, length, and field coverage in *trio^RNAi^* relative to controls. *trio* knockdown results in a significant reduction in the number of dendritic terminals reflecting a decrease in overall branching (**C**) and a reduction in total dendritic length (**D**). (**E**) Disruption of *trio* function also leads to a reduction in the average dendritic length per branch. (**F**) The percentage of dendritic field coverage is significantly reduced with *trio* knockdown (71%) as compared to controls (95%) reflecting defects in branching and growth. (**G**) Analyses of dendritic branch order reveal defects in the specification of higher order branching resulting in a proximal shift in branch order distribution in *trio* knockdown (*n* = 7) relative to controls (*n* = 6). Images were taken at 20× magnification and size bar represents 50 microns. The total *n* value for each neuron and genotype quantified is reported on the bar graph. Statistically significant *p* values are reported on the graph as follows (** = *p*<0.01; *** = *p*<0.001). Genotypes: **WT**: *GAL4^477^,UAS-mCD8::GFP/+*. ***trio^RNAi^***: *GAL4^477^,UAS-mCD8::GFP/+;UAS-trio^RNAi^/+*.

Class IV neurons represent the most complex class of da neurons characterized by space-filling dendritic arbors exhibiting highly complex branching morphology and nearly complete dendritic field coverage [40]. Qualitative analyses of *trio* knockdown in class IV ddaC neurons revealed a reduction in dendritic branch complexity relative to control neurons (Fig. 4A, 4B). This phenotype was verified by quantitative comparisons which revealed a significant reduction in both the number of dendritic terminals (Fig. 4C) and total dendritic length (Fig. 4D). In contrast, disruption of *trio* function results in an increase in the average dendritic length per branch relative to controls which is consistent with the overall reduction in dendritic branch complexity (Fig. 4E). Analyses of the percentage of dendritic field coverage revealed a significant reduction in *trio* knockdown (71% field coverage) as compared to controls (95% field coverage) indicating *trio* function is essential for establishing full dendritic field coverage (Fig. 4F). Dendritic branch order analyses of ddaC neurons reveals a proximal shift in *trio* knockdown relative to controls reflecting a higher percentage of lower order branches and a reduction in the percentage of higher order branches (Fig. 4G). These results demonstrate that Trio functions cell-autonomously in promoting higher order branching and field coverage in class IV neurons.

### Trio sculpts dendritic morphology via interactions with Rac1 and Rho1

**Figure 5:**
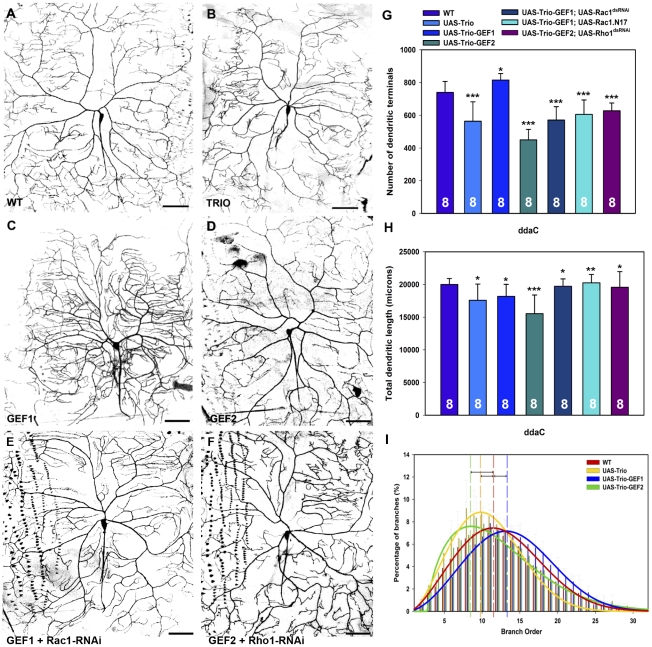
Trio overexpression in class IV da neurons. (**A–F**) Live confocal images of third instar larval dorsal ddaC class IV da neurons labeled with *GAL4^477^,UAS-mCD8::GFP*. As compared to control (**A**), Trio overexpression results in decreased dendritic branching (**B**). (**C**) In contrast, Trio-GEF1 overexpression leads to an increase in dendritic branching, whereas Trio-GEF2 overexpression results in a reduction in dendritic branching (**D**). (**E**) RNAi knockdown of Rac1 in a Trio-GEF1 overexpression background suppresses defects in dendritic development as compared to Trio-GEF1 overexpression alone. (**F**) RNAi knockdown of Rho1 in a Trio-GEF2 overexpression background suppresses defects in dendrite morphogenesis as compared to Trio-GEF2 overexpression alone. (**G**) Analyses of the number of dendritic terminals reveals a decrease in dendritic branching with Trio and Trio-GEF2 overexpression whereas Trio-GEF1 overexpression leads to an increase in dendritic branching relative to wild-type controls. Knockdown of Rac1 via RNAi or by co-expression of the dominant negative Rac1.N17 suppresses defects in dendritic branching relative to Trio-GEF1 overexpression alone, whereas knockdown of Rho1 via RNAi suppresses defects in branching as compared to Trio-GEF2 overexpression alone. (**H**) Quantitation of total dendritic length reveals a mild to moderate reduction with Trio, Trio-GEF1 and Trio-GEF2 overexpression as compared to wild-type controls. Consistent with dendritic branching, disrupting Rac1 or Rho1 function suppresses defects in dendritic length as compared to Trio-GEF1 or Trio-GEF2 overexpression, respectively. (**I**) Relative to control (*n* = 6), dendritic branch order analyses reveal a proximal shift in the percentage of lower order branching with Trio (*n* = 8) and Trio-GEF2 (*n* = 8) overexpression, whereas Trio-GEF1 (*n* = 8) overexpression results in a distal shift towards higher order branching in class IV ddaC neurons. Images were taken at 20× magnification and size bar represents 50 microns. The total *n* value for each neuron and genotype quantified is reported on the bar graph. Statistically significant *p* values are reported on the graphs as follows (* = *p*<0.05; ** = *p*<0.01; *** = *p*<0.001). Genotypes: **WT**: *GAL4^477^,UAS-mCD8::GFP*/+. **TRIO**: *UAS-trio/+*;*GAL4^477^,UAS-mCD8::GFP/+*. **GEF1**: *UAS-trio-GEF1-myc/GAL4^477^,UAS-mCD8::GFP.* **GEF2**: *GAL4^477^,UAS-mCD8::GFP/+;UAS-trio-GEF2-myc/+*. **GEF1+Rac1-RNAi**: *UAS-trio-GEF1-myc/GAL4^477^,UAS-mCD8::GFP;UAS-Rac1^JF02813^*/+. **GEF1+Rac1.N17**: *UAS-trio-GEF1-myc/GAL4^477^,UAS-mCD8::GFP;UAS-Rac1.N17*/+ **GEF2+Rho1-RNAi**: *GAL4^477^,UAS-mCD8::GFP/+;UAS-trio-GEF2-myc/UAS-Rho1-dsRNA*.

To further explore Trio function in directing class specific dendrite morphogenesis, we conducted gain-of-function studies in class I, III, and IV da neurons. We initiated our overexpression analyses in class IV ddaC neurons. Phenotypic analyses revealed defects in dendritic branching morphology, including a reduction in branching proximal and distal to the cell body, with full length Trio overexpression relative to wild-type controls (Fig. 5A, 5B). This observation was confirmed by significant decreases in the number of dendritic terminals (Fig. 5G) and overall dendritic length (Fig. 5H). However, analyses of average dendritic length per branch failed to reveal any significant change (Fig. S1A). To determine how Trio overexpression may effect dendritic field coverage, we quantified the percentage of total field coverage which revealed a significant decrease (81% field coverage) relative to controls (95% field coverage) (Fig. S1B). Dendritic branch order analyses of ddaC neurons revealed a proximal shift in branch order distribution with Trio overexpression resulting in a higher percentage of lower order branching overall relative to controls (Fig. 5I).

As Trio contains two independent GEF activation domains, the Rac1-specific GEF1 domain and the Rho1-specific GEF2 domain, we sought to dissect the relative contributions of each of these domains to the regulation of class IV dendrite morphogenesis. We independently overexpressed *UAS-trio-GEF1-myc* and *UAS-trio-GEF2-myc* and verified expression of these Myc-tagged transgenes in class IV neurons (Fig. S2). These analyses demonstrate that both transgenes are strongly expressed in class IV ddaC neurons, however quantitative analyses reveal differential expression levels between the two transgenes, with a mild, but significantly higher level of Myc expression observed with the *UAS-trio-GEF1-myc* transgene relative to the *UAS-trio-GEF2-myc* transgene (Fig. S2I). Given that the two transgenes are inserted at independent sites within the genome, we propose that the differences in expression levels are likely due to position effects.

Overexpression of Trio-GEF1 led to a dramatic increase in dendritic branching complexity particularly near the cell body and a concomitant reduction in dendritic extension toward the dorsal midline and lateral hemisegment boundaries (Fig. 5C). These phenotypes were verified by quantitative analyses which revealed a significant increase in dendritic branching (Fig. 5G), a moderate reduction in overall dendritic length (Fig. 5H) and a sharp decrease in the average length per dendritic branch (Fig. S1A). Analyses of the dendritic field coverage revealed a strong reduction with Trio-GEF1 overexpression (70% field coverage) relative to control (95% field coverage) (Fig. S1B). This data is consistent with the reduction in overall dendritic length as well as with the qualitative phenotypic data in which dendritic branching was concentrated proximal to the cell body. Moreover, analyses of dendritic branch order in ddaC neurons demonstrate that Trio-GEF1 overexpression results in a distal shift in branch order distribution towards a greater percentage of higher order branching as compared to control (Fig. 5I). In contrast, Trio-GEF2 overexpression caused a strong reduction in dendritic branching complexity (Fig. 5D) which was reflected in the significant reductions in the number of dendritic terminals (Fig. 5G), total dendritic length (Fig. 5H) and increase in the average length per dendritic branch (Fig. S1A). Moreover, Trio-GEF2 overexpression likewise caused a significant reduction in dendritic field covereage (79% field coverage) as compared to control (95% field coverage) (Fig. S1B). In addition, dendritic branch order analyses indicate that Trio-GEF2 overexpression results in a proximal shift in branch order distribution giving rise to a higher percentage of lower order branching relative to control (Fig. 5I). Finally, we compared whether or not co-overexpression of Trio-GEF1 and Trio-GEF2 could potentially phenocopy the effects on class IV dendritogenesis observed with full length Trio overexpression (Fig. S3). These analyses revealed no significant differences with respect to the number of dendritic terminals or total dendritic length (data not shown), however qualitatively the phenotypes did not appear to be precise phenocopies (Fig. S3A, S3B). As such, we also examined branch order distribution and found that phenotypically, co-overexpression of the GEF1 and GEF2 domains yields a distal shift to an increased percentage of higher order branching in class IV dendrites as compared to full length Trio overexpression (Fig. S3C). This phenotype is consistent with our observations that the Trio-GEF1 transgene expresses at higher levels compared to the Trio-GEF2 transgene and as such we predicted that the co-overexpression phenotype would likely be more similar to the GEF1 overexpression phenotype.

Based on previous studies [17]–[21], [24], [25], we hypothesized that the Trio GEF1 domain functions in the activation of Rac1 and the GEF2 domain in activation of Rho1 in da neurons. To directly address this question, we conducted phenotypic epistasis experiments to validate interactions between Trio-GEF1/Rac1 and Trio-GEF2/Rho1 in da neurons. For these studies, we simultaneously overexpressed the GEF1 or GEF2 domains and RNAi knockdown transgenes for Rac1 or Rho1, respectively, in class IV da neurons. Phenotypic analyses revealed that Rac1 knockdown suppresses Trio-GEF1 induced defects in dendritic branching (Fig. 5E), whereas Rho1 knockdown suppresses dendritic defects observed with Trio-GEF2 overexpression (Fig. 5F). Consistent with this, quantitative analyses demonstrate that Rac1 and Rho1 knockdown significantly suppress defects in dendritic branching (Fig. 5G) and length (Fig. 5H) relative to Trio-GEF1 and Trio-GEF2, respectively. In addition, we demonstrated that expression of a dominant negative Rac1 likewise suppresses defects in dendritic branching (Fig. 5G) and length (Fig. 5H) relative to Trio-GEF1 overexpression alone. These results confirm that Trio-GEF1 functions through Rac1 to promote dendritic branching and Trio-GEF2 functions through Rho1 to inhibit dendritic branching. Moreover the opposing effects of Rac1 versus Rho1 activation may account for the intermediate effects observed with overexpression of full length Trio as compared to that of GEF1 or GEF2 alone.

**Figure 6:**
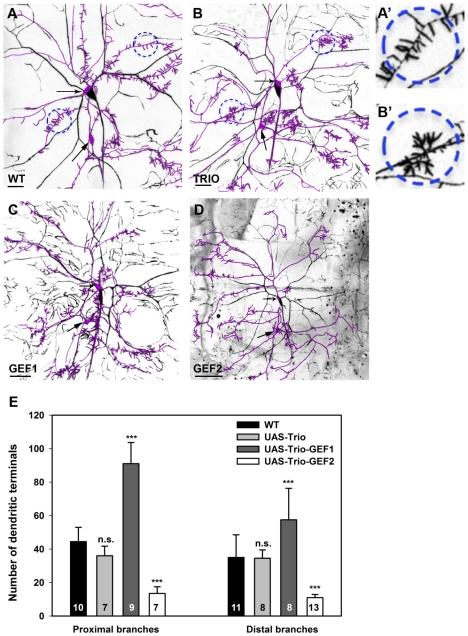
Trio overexpression in class III da neurons. (**A–D**) Live confocal images of class III and IV dorsal cluster da neurons at the third larval instar labeled with the F-actin reporter *UAS-GMA* driven by the *ppk-GAL4* transgene. Class III ddaA and ddaF neuron cell bodies are indicated by arrows and for clarity the class III neuron cell bodies and dendrites have been highlighted by a magenta pseudo-color trace overlay. (**A**) Representative image of wild-type class III da neuron displaying regularly distributed, unbranched, actin-rich dendritic filopodia (dashed line circles and high magnification image (**A′**)) projecting from the primary dendritic branches. (**B**) Full length Trio overexpression dramatically altered dendritic filopodia producing a hyper-branched, splintered morphology (dashed line circles and high magnification image (**B′**)). In addition, the filopodia displayed a more clustered distribution as compared to control. (**C**) Overexpression of the Trio-GEF1 domain increased dendritic branching overall and produced a highly similar splintered morphology on dendritic filopodia. (**D**) Overexpression of the Trio-GEF2 domain produces the opposite effect by reducing dendritic branching overall and leading to a dramatic decrease in the number of dendritic filopodia. (**E**) Quantitative analyses of the number of dendritic terminals as a measure of dendritic branching reveals no significant change with full length Trio overexpression, whereas upregulation of Trio-GEF1 dramatically leads to a significant increase in terminals both proximal and distal to the cell body, while upregulation of Trio-GEF2 leads to a significant decrease in terminals both proximal and distal to the cell body. The total *n* value for each neuron and genotype quantified is reported on the bar graph. Statistically significant *p* values are reported on the graph as follows (n.s. = not significant; *** = *p*<0.001). Genotypes: **WT**: *UAS-GMA/+;ppk-GAL4*/+;+. **TRIO**: *UAS-GMA/UAS-trio;ppk-GAL4/+*;+. **GEF1**: *UAS-GMA/+;ppk-GAL4*/*UAS-trio-GEF1-myc*;+. **GEF2**: *UAS-GMA/+;ppk-GAL4*/*+*;*UAS-trio-GEF2-myc/*+.

To determine if these Trio gain-of-function effects are observed in other complex da neuron subclasses, we examined overexpression in class III da neurons. Qualitative phenotypic analyses revealed that full length Trio overexpression primarily affects the characteristic class III dendritic filopodia which normally exist as single, actin-rich processes extending from primary branches, however following upregulation of Trio these individual filopodia display a highly branched or “splintered” morphology and do not appear as evenly distributed along the primary dendritic shafts (Fig. 6A, 6A′, 6B, 6B′). Quantitatively, however, we observed no statistically significant difference with respect to the number of dendritic branch terminals either proximal or distal to the cell body in Trio overexpressing class III neurons relative to controls (Fig. 6E). Taken together, these data indicate that while overexpression of full length Trio alters branching morphology and distribution of dendritic filopodia there is no overt change in the number of dendritic termini.

Consistent with our observations in class IV neurons, overexpression of the Trio-GEF1 domain resulted in a similar effect on increasing overall dendritic branching, and in the case of class III neurons phenocopied the splintered filopodial phenotype observed with full length Trio overexpression (Fig. 6C). These data suggest that the Trio-GEF1 mediated activation of Rac1 is likely responsible for the increased branching in filopodia and is consistent with previous reports documenting the same phenotype following Rac1 overexpression in class III neurons [21]. Quantitative analyses confirm that Trio-GEF1 upregulation contributes to a significant increase in dendritic branching both proximal and distal to the cell body (Fig. 6E). In sharp contrast, Trio-GEF2 overexpression sharply reduces dendritic branching and leads to a marked decrease in the formation of dendritic filopodia (Fig. 6D, 6E). Collectively, these data suggest that Trio-GEF1 activation of Rac1 promotes, while Trio-GEF2 activation of Rho1 inhibits branching in class III dendrites, particularly actin-rich dendritic filopodia. The opposing effects of GEF1 versus GEF2 on dendrite morphogenesis could also potentially explain the observed effects with full length Trio overexpression in which both the GEF1 and GEF2 domains are simultaneously upregulated.

**Figure 7:**
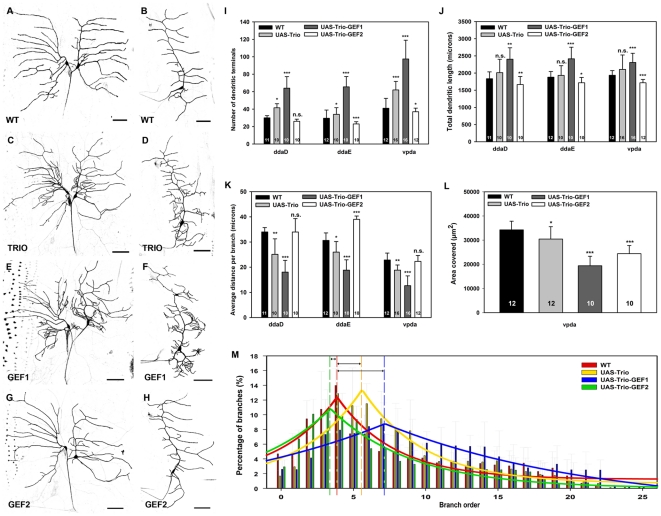
Trio overexpression in class I da neurons. (**A–H**) Live confocal images of third instar larval dorsal (ddaD/E) and ventral (vpda) class I da neurons labeled with *GAL4^221^,UAS-mCD8::GFP*. As compared to controls (**A,B**), Trio overexpression results in increased dendritic branching in ddaD/E (**C**) and vpda (**D**) class I neurons. Trio-GEF1 overexpression likewise leads to a dramatic increase in dendritic branching in ddaD/E (**E**) and vpda (**F**) neurons. In contrast, Trio-GEF2 overexpression results in a mild reduction in dendritic branching in ddaD/E (**G**) and (**H**) vpda neurons. (**I–M**) Quantitative analyses of dendritic branching, length, and field coverage in Trio, Trio-GEF1, and Trio-GEF overexpression relative to controls. (**I**) Analyses of the number of dendritic terminals reveals an increase in dendritic branching in all class I neurons following Trio and Trio-GEF1 overexpression, whereas Trio-GEF2 overexpression leads to a reduction in branching in ddaE and vpda neurons. (**J**) Quantitation of total dendritic length reveals no significant change following Trio overexpression, however with Trio-GEF1 overexpression there is an increase in length as a function of higher levels of dendritic branching, whereas there is a decrease in length with Trio-GEF2 overexpression. (**K**) The average length per dendritic branch is significantly reduced in both Trio and Trio-GEF1 overexpression, whereas Trio-GEF2 overexpression leads to an increase in ddaE neurons. (**L**) Overexpression of Trio, Trio-GEF1, and Trio-GEF reduces total dendritic area of vpda neurons as compared to control. (**M**) Relative to control (*n* = 12), dendritic branch order analyses in vpda neurons reveals a distal shift in the percentage of higher order branching with Trio (*n* = 16) and Trio-GEF1 (*n = *16) overexpression, whereas a slight proximal shift towards lower order branching, with a steep decline in higher order branching, is observed with Trio-GEF2 overexpression (*n* = 12). Images were taken at 20× magnification and size bar represents 50 microns. The total *n* value for each neuron and genotype quantified is reported on the bar graph. Statistically significant *p* values are reported on the graphs as follows (* = *p*<0.05; ** = *p*<0.01; *** = *p*<0.001; n.s. = not significant). Genotypes: **WT**: *GAL4^221^,UAS-mCD8::GFP*/+. **TRIO**: *UAS-trio/+*;+; *GAL4^221^,UAS-mCD8::GFP/+*. **GEF1**: *UAS-trio-GEF1-myc/+*;*GAL4^221^,UAS-mCD8::GFP/*+. **GEF2**: *UAS-trio-GEF2-myc/GAL4^221^,UAS-mCD8::GFP*.

In light of the variable effects observed in the more complex class III and IV da neurons, we examined the potential effects of Trio overexpression in the morphologically simple class I da neurons. Trio overexpression increased dendritic branching in all class I da neurons relative to wild-type controls (Fig. 7A–D), resulting in a significant increase in the number of dendritic terminals reflecting an increase in overall branching (Fig. 7I). Interestingly, the increase in dendritic branching did not contribute to a significant change in total dendritic length (Fig. 7J). Moreover, analyses of the average dendritic length per branch revealed a significant decrease with Trio overexpression as compared to controls (Fig. 7K) suggesting that the increased branching observed was restricted to higher order fine terminal branches along with a decrease in primary branch extension. Moreover, we found the Trio overexpression reduced the total dendritic area covered by class I vpda neurons as compared to control which appears to be a consequence of the increase in short, higher order branching emanating from the primary branches (Fig. 7L). Dendritic branch order analyses confirm that Trio overexpression in class I vpda neurons promotes a distal shift in branch order distribution towards a greater percentage of higher order branches relative to control (Fig. 7M).

Consistent with full length Trio, Trio-GEF1 overexpression led to a dramatic increase in dendritic branching in all class I neurons (Fig. 7E, 7F). Quantitative analyses revealed a significant increase in dendritic branching with Trio-GEF1 overexpression that is even more robust than that observed with full length Trio overexpression (Fig. 7I). In contrast to full length Trio, overexpression of Trio-GEF1 also results in an increase in overall total dendritic length (Fig. 7J). In addition, we observed a strong reduction in average dendritic branch length (Fig. 7K) which is consistent with the observation of increased fine terminal branching relative to controls and suggests that the increase in total dendritic length is due to the strong increase in the formation of *de novo* dendritic branches. With respect to total dendritic area, GEF1 overexpression results in a significant decrease in total area as a function of decreased extension coupled with the increased incidence of clustered, higher order branching (Fig. 7L). Moreover, consistent with the effects observed with Trio overexpression, dendritic branch order analyses of vpda neurons reveals a strong distal shift towards in the percentage of higher order branching with GEF1 overexpression relative to control (Fig. 7M). In sharp contrast, overexpression of Trio-GEF2 led to a reduction in dendritic branching (Fig. 7G, 7H). Upregulation of GEF2 activity results in a significant reduction in dendritic branching in both ddaE and vpda class I neurons (Fig. 7I) as well as an overall reduction in total dendritic length in all class I neurons (Fig. 7J). Moreover, increased GEF2 activity led to a significant increase in average dendritic length per branch in ddaE neurons as compared to controls (Fig. 7K), whereas no significant change was observed in ddaD or vpda neurons. This increase in average dendritic length per branch is likely a function of the reduction in dendritic branching observed with GEF2 overexpression. In terms of total dendritic area, GEF2 overexpression results in a reduction in area (Fig. 7L) due to the reduction in overall dendritic branching and length. Analyses of dendritic branch order indicates that GEF2 overexpression leads to a proximal shift in branch order distribution towards a higher percentage of lower order branching and a sharper decline in the percentage of higher order branches relative to controls (Fig. 7M). Collectively, these analyses suggest that the effects on dendritic branching observed with full length Trio overexpression likely result from the opposing effects of Rac1 and Rho1 activation contributing to sculpting of overall dendritic branching and extension. Moreover, these results are consistent with those observed in class III and IV neurons in which Trio-GEF1 interaction with Rac1 promotes dendritic branching and Trio-GEF2 interaction with Rho1 inhibits dendritic branching.

### Cut induced *de novo* dendritic branching and filopodia formation require Trio activity

**Figure 8:**
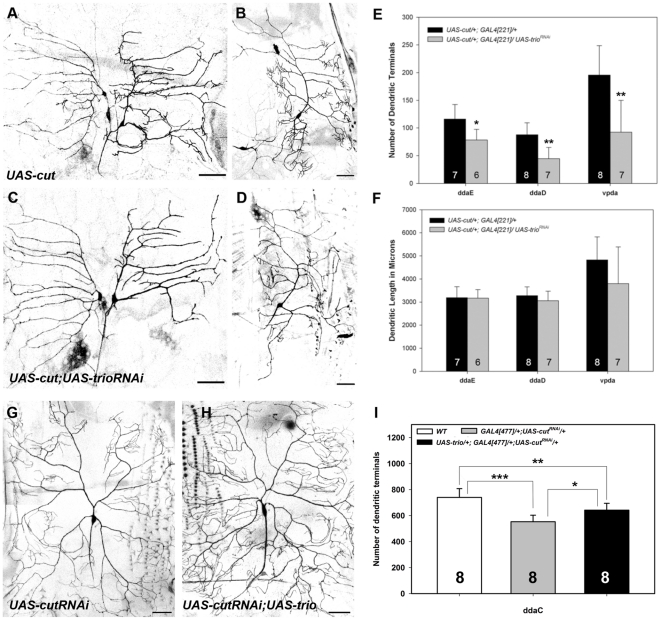
Cut-induced *de novo* dendritic branching and filopodia formation requires Trio activity. (**A–D**) Live confocal images of class I da neurons (ddaD, ddaE, vpda) at the third larval instar. (**G,H**) Live confocal images of class IV ddaC neurons. Images were collected at 20× magnification and size bars represents 50 microns. (**A,B**) Representative images of dorsal ddaD/E neurons (**A**) and ventral vpda neuron (**B**) ectopically overexpressing Cut. Cut ectopic overexpression results in class I neurons displaying increased dendritic branching complexity characterized by a high incidence of dendritic filopodia emanating from the primary branches. (**C,D**) Representative images of dorsal ddaD/E neurons (**C**) and ventral vpda neuron (**D**) in which Cut ectopic overexpression is combined with knockdown of *trio* via *UAS-trio^RNAi^*. As compared to Cut overexpression, *trio* knockdown results in strong suppression of the Cut phenotype, particularly with respect to dendritic filopodia. (**E**) Quantitative analyses reveal a significant reduction in the total number of dendritic terminals in neurons expressing *trio^RNAi^* relative to Cut ectopic overexpression alone. (**F**) Quantitative analyses reveal no statistically significant difference in total dendritic length. (**G**) Representative image of class IV ddaC neuron expressing *UAS-cut^RNAi^*. (**H**) Representative image of ddaC neuron simultaneously expressing *UAS-cut^RNAi^* and *UAS-trio* transgenes reveals partial rescue of *cut* mutant defects in dendritic branching. (**I**) Quantitative analyses reveal knockdown of *cut* via RNAi significantly reduces the total number of dendritic terminals relative to wild-type (WT) controls, whereas Trio overexpression in a *cut^RNAi^* background partially rescues the *cut* mutant phenotype. The total *n* value for each neuron and genotype quantified is reported on the bar graph. Statistically significant *p* values are reported on the graph as follows (* = *p*<0.05; ** = *p*<0.01; *** = *p*<0.001). Genotypes: (**A,B**) *UAS-cut/+; GAL4^221^,UASmCD8::GFP/+*; (**C,D**) *UAS-cut/+; GAL4^221^,UASmCD8::GFP/UAS-trio^RNAi^*; (**G**) *GAL4^477^,UASmCD8::GFP/+;UAS-cut^RNAi^/+*; (**H**) *UAS-trio/+; GAL4^477^,UASmCD8::GFP/+; UAS-cut^RNAi^/+*.

The Cut homeodomain transcription factor has previously been demonstrated to exert class specific effects on da neuron dendrite morphology where high levels of Cut expression are correlated with more complex patterns of da neuron dendritic arborization [42]. In addition, ectopic overexpression of Cut in class I da neurons was shown to dramatically alter dendritic arborization characterized by increased dendritic length, branching, and the development of numerous spine-like dendritic filopodia similar to those normally observed in class III da neurons [42]. Moreover, Cut has been shown to synergistically interact with Rac1 in promoting *de novo* actin-rich dendritic filopodia formation in class I da neurons [23]. As we have demonstrated that disruptions in *trio* function led to reduced dendritic branching complexity in da neurons, whereas overexpression of Trio and the Rac1-specific GEF1 domain led to increased dendritic complexity and *de novo* formation of actin-rich dendritic filopodia, we hypothesized that Trio may function downstream of Cut in mediating dendritic branching complexity and the formation of these filopodial processes. We reasoned that if the Cut ectopic overexpression phenotype requires Trio function as a downstream effector then disruptions in Trio activity may result in a suppression of Cut-mediated changes in dendritic morphology. To address this, we compared class I da neurons ectopically overexpressing Cut in the presence or absence of *trio^RNAi^* to determine whether knockdown of *trio* could suppress the formation of these dendritic filopodia. These analyses revealed strong suppression of the Cut overexpression phenotype in the presence of *trio^RNAi^* as compared to controls (Fig. 8A–D). Quantitative analyses of Trio-mediated suppression was statistically significant with respect to the number of dendritic terminals (Fig. 8E), however no statistically significant difference was observed in overall dendritic length (Fig. 8F) as compared to controls. The predominant phenotypic suppression observed following *trio* knockdown was a strong reduction in the number of dendritic filopodia emanating from the primary branches.

To further explore the putative regulatory relationship between Cut and Trio, we performed a related set of studies in class IV da neurons in which Cut is normally expressed. We hypothesized that if Cut acts via Trio in mediating da neuron dendritogenesis, then Trio overexpression in a *cut* mutant background may potentially rescue *cut-*induced dendrite morphogenesis defects. For these analyses, we compared class IV ddaC neurons expressing a *UAS-cut^RNAi^* transgene in the presence or absence of a full length *UAS-trio* transgene. Relative to wild-type controls, expression of *UAS-cut^RNAi^* in class IV neurons produced a significant reduction in dendritic branching complexity (Fig. 8G, 8I) consistent with previous findings [42]. In contrast, expression of full length Trio in the *cut^RNAi^* background produced a partial, but significant rescue of dendritic branching complexity providing additional evidence that Trio functions downstream of Cut in mediating class-specific da neuron dendrite morphogenesis (Fig. 8H, 8I).

Given that Trio is normally expressed in class I da neurons, whereas Cut is not normally expressed, we can conclude that Trio expression is not solely dependent upon Cut transcriptional regulation. To determine whether any potential transcriptional regulatory relationship between Cut and Trio exists in other da neuron subclasses where Cut is normally expressed (class II–IV), we performed MARCM analyses using a *cut* null allele and stained mutant clones for Trio expression. These analyses confirmed that Cut is not absolutely required for Trio expression in da neurons (data not shown). Although Cut is not essential for Trio expression, we investigated whether Cut overexpression could upregulate Trio expression in da neurons. For these analyses, we ectopically overexpressed Cut in class I da neurons and then labelled control and experimental filets for Trio. Qualitative staining suggested that Cut may be mildly upregulating Trio expression, therefore we performed quantitative analyses of relative fluorescence intensities for Trio in Cut overexpressing neurons and control neurons. Moreover, we normalized this data for experimental and control by measuring Trio fluorescence intensity in adjacent class III da neurons which were not overexpressing Cut. We found a mild, but highly significant upregulation of Trio expression in Cut overexpressing class I neurons (∼10%) relative to control Trio levels in the absence of Cut overexpression (Fig. S4). Collectively, these data indicate that while Cut is not absolutely required for Trio expression, Cut can upregulate Trio in da neurons.

Finally, to determine if Trio can synergistically interact with Cut in promoting dendritic branching and the formation of actin-rich dendritic filopodia as previously demonstrated between Cut and Rac1 [23], we examined the effects of co-expression of Cut and Trio in class I neurons. Phenotypic analyses indicate that co-expression of Cut and full length Trio result in a moderate increase in dendritic branching (Fig. S5B, S5E) and dendritic length (Fig. S5B, S5F) relative to Cut ectopic overexpression alone (Fig. S5A, S5E, S5F), whereas co-expression of Cut and the Trio-GEF1 domain produce a strong phenotypic increase in total dendritic terminals resulting in a concomitant increase in total dendritic length (Fig. S5C, S5E, S5F). In contrast, we found that co-expression of Cut and the Trio-GEF2 domain produced no significant change in total dendritic terminals (Fig. S5D, S5E), whereas a strong increase in dendritic extension was observed resulting in increased total dendritic length (Fig. S5D, S5F) as compared to Cut ectopic overexpression alone. These results indicate that Cut can synergistically interact with Trio in regulating dendritogenesis, whereby the GEF1 domain acts in promoting dendritic branching and the GEF2 domain acts to promote dendritic extension.

## Discussion

Collectively, our analyses demonstrate that Trio functions in promoting and refining class specific dendritic arborization patterns via GEF1- and GEF2-dependent interactions with Rac1 and Rho1, respectively. We also demonstrate that Trio is required in mediating Cut induced effects on dendritic branching and filopodia formation suggesting that these molecules may operate in a common pathway to direct dendritic morphogenesis. Moreover, during the preparation of these studies for publication we became aware that Dr. Edward Giniger and colleague (NINDS/NIH) had likewise been investigating Trio function in da neurons via a non-overlapping, complementary experimental approach and that they arrived at conclusions regarding Trio function largely consistent with those reported here.

Previous studies have demonstrated that Trio functions via its GEF1 domain in mediating the regulation of axon morphogenesis by modulating Rac1 activity [28], [30], [36], however much less is known regarding the potential *in vivo* functional role(s) of the Trio GEF2 domain. Intriguingly, a previous study demonstrated that *trio* mutant neuroblast clones display a neurite overextension phenotype from the dendritic calyx region of the mushroom body [27] which strongly resembled the dendrite-specific overextension phenotype observed in *RhoA* mutant mushroom body clones [16] suggesting that RhoA/Rho1 activation may be required for restricting dendritic extension. In *Drosophila* da neurons, *trio* loss-of-function analyses reveal a reduction in dendritic branching in three distinct da neuron subclasses (class I, III, and IV), indicating a functional role for Trio in promoting dendritic branching. However, class specific differences are observed with Trio gain-of-function studies in which Trio overexpression in class I neurons increases dendritic branching, whereas in class III neurons there is no change in overall dendritic branching, but rather a redistribution of branches, and in class IV there is a reduction in overall dendritic branching. The basis for these differences appear to lie in our observation that refinement of dendritic branching in da neurons is subject to the opposing roles of Rac1 and Rho1 activation via Trio-GEF1 and Trio-GEF2, respectively, where Trio-GEF1 activity promotes higher order dendritic branching, whereas Trio-GEF2 activity restricts higher order branching and also limits overall dendritic length/extension.

One of the key distinctions between class I versus class III and IV neurons relates to inherent differences in normal dendritic branching complexity and the relative roles of dynamic actin cytoskeletal based processes in these neurons which are known to mediate higher order branching including the dendritic filopodia of class III neurons and fine terminal branching in class IV neurons [21], [23], whereas the class I neurons do not normally exhibit this degree of higher order branching and are predominantly populated by stable, microtubule-based primary and secondary branches [23]. As such, Trio overexpression in these distinct subclasses may yield different effects on overall dendritic branching morphology based upon the normal distribution of actin cytoskeleton within these subclasses leading to unique effects on class specific dendritic architecture. Both loss-of-function and gain-of-function results support this hypothesis as the predominant effects are restricted to actin-rich higher order branching, whereas the primary branches populated by microtubles are relatively unaffected. This is further supported by the demonstration that *trio* knockdown suppresses Cut induced formation of actin-rich dendritic filopodia. Moreover, phenotypic analyses revealed that co-expression of Cut and Trio-GEF1 synergistically enhance dendritic branching in class I neurons likely due to increased activation of Rac1, whereas co-expression of Cut and Trio-GEF2 lead primarily to increased dendritic extension likely due to increased activation of Rho1. Thus, Trio mediated regulation of Rac1 and/or Rho1 signaling has the potential for sculpting dendritic branching and outgrowth/extension depending upon the combinatorial and opposing effects of Rac1 and Rho1.

In contrast to Cut, which has been shown to be differentially expressed in da neuron subclasses and exert distinct effects on class specific dendritic arborization [42], we have demonstrated that Trio is expressed in all da neuron subclasses and can exert distinct effects on class specific dendritic branching. For example, in all subclasses examined, loss-of-function analyses indicate Trio is required to promote dendritic branching and yet individual subclasses exhibit strikingly distinct dendritic morphologies. These results suggest that Trio is generally required in each of these subclasses to regulate branching, however alone is insufficient to drive these class specific morphologies solely via activation of Rac1 and/or Rho1 signaling. One logical hypothesis is that differential expression of RhoGAP family members in distinct da neuron subclasses may work in concert with Trio to refine class specific morphologies. The potential for combinatorial activity between Trio and various RhoGAPs is significant given that 20 RhoGAPs have been defined in the *Drosophila* genome [43]. For example, given that class I da neurons exhibit a simple branching morphology which becomes more complex when Trio or Trio-GEF1 domains are overexpressed, perhaps there is higher expression of Rac-inactivating GAPs in class I neurons that function in limiting dendritic branching, whereas in the more complex class III or IV da neurons, there may be lower expression of RacGAPs. Since overexpression of Trio-GEF2 reduces dendritic branching complexity in all three da neuron subclasses we analyzed, one might predict that Rho1 activation limits dendritic branching and that therefore the expression of RhoGAPs may be modulated to facilitate branching in class III and IV neurons relative to class I neurons. In concert, differential expression of RacGAPs and RhoGAPs together with the uniform expression of Trio in all da neuron subclasses could potentially account for differential levels of activation/inactivation of Rac1 and/or Rho1 in individual subclasses and thereby influence overall class specific dendritic architecture.

In support of this hypothesis, class-specific microarray analyses conducted in class I, III, and IV da neurons indeed reveal differential gene expression levels for most of the 20 known RhoGAP family members at a class-specific level (Iyer, Iyer, and Cox, unpublished data). These expression analyses reveal one trend whereby select RhoGAP encoding genes are upregulated in the more complex class III and IV da neurons relative to the simple class I da neurons, whereas select RacGAP encoding genes are downregulated in complex neurons relative to simple neurons. Moreover, it is known that individual RhoGAPs display differential specificities for Rac, Rho and Cdc42 *in vivo* [44], such that a given RhoGAP may function in activating one or more of these small G proteins thereby increasing the potential for fine-tuning activation levels of a particular G protein at a class specific level. Furthermore recent studies provide direct evidence of the importance of RhoGAP family members in regulating da neuron dendritic morphogenesis. Analyses of the *tumbleweed* (*tum*) gene, which encodes the GTPase activating protein RacGAP50C, demonstrate that *tum* mutants display excessive da neuron dendritic branching [18], [45], [46]. The dendritic phenotype observed in *tum* mutant da neurons is strikingly similar to that observed with Trio-GEF1 overexpression which we demonstrate also leads to excessive dendritic branching. Together these data suggest that Trio-GEF1 functions in activating Rac1 to promote dendritic branching whereas Tum/RacGAP50C function in inactivating Rac1 via its GTPase activity and thereby limit dendritic branching. In contrast, mutant analyses of the RhoGAP encoding gene, *crossveinless-c*, whose target in da neurons is the Rho1 small G protein, reveal defects in directional growth of da neuron dendrites [17]. These results indicate that Crossveinless-C is required to inactivate Rho1 in order to promote directional dendritic growth and further suggest that a failure to inactivate Rho1 leads to restricted dendritic growth consistent with the phenotypes we observed with Trio-GEF2 overexpression in all da neuron subclasses examined. These results, together with those presented herein, suggest that potential combinatorial activity of Trio and RhoGAP family proteins may converge in shaping the class specific dendritic architecture. Ultimately, future functional studies will be required to validate this hypothesis.

While previous studies have revealed Trio acts in concert with Abl and Ena in coordinately regulating axon guidance [28], [29], [47], the same regulatory relationship does not appear to operate in da neuron dendrites as Abl has been shown to function in limiting dendritic branching and the formation of dendritic filopoda, whereas both Ena functions in promoting dendritic branching [48]. We demonstrate that Trio functions in promoting dendritic branching, consistent with Ena activity, but in da neuron dendrites works in an opposite direction to Abl. These findings suggest that, at least in da neuron dendrites, Trio may operate in either an Abl-independent pathway or that Trio and Abl may exhibit a context dependent regulatory interaction that is distinctly different in dendrites versus axons.

## Materials and Methods

### *Drosophila* strains

*Drosophila* strains used in this study were raised on standard cornmeal-molasses-agar media at 25°C unless otherwise noted. Fly strains were obtained from Bloomington (*GAL4^477^*,*UAS-mCD8::GFP*; *UAS-trio.B* [28]; *UAS-trio-GEF1-myc* [30]; *UAS-trio-GEF2-myc* [30]; *UAS-trio^JF02815^*; *UAS-Rac1^JF02813^; UAS-Rac1.N17* [49]; *UAS-Rho1-dsRNA; UAS-cut^JF03304^*), Vienna *Drosophila* RNAi Center (*UAS-trio^GD9531^*) and other sources (*GAL4^ppk.1.9^, UASmCD8::GFP* [50]; *w; ppk-GAL4,UASmCD8GFP*; *GAL4^221^,UASmCD8::GFP* [42]; *GAL4^21-7^, UAS-mCD8::GFP* [51]; *UAS-GMA* [43]; *UAS-cut*; *w,ct^c145^,FRT^19A^/FM7/y^+^,ct^+^,Y*; *y,w,tubP-GAL80,hsFLP,FRT^19A^; GAL4^109(2)80^, UAS-mCD8::GFP* [42]). *Oregon-R* was used as a wild-type strain. To enhance expression, crosses involving *GAL4/UAS* combinations were reared at 29°C for both control and experimental backgrounds.

### Immunohistochemistry

Third instar larval filet dissection and immunohistochemistry (IHC) was performed essentially as previously described [52]. Primary antibodies used in this study include: mouse anti-Trio (9.4A; 1∶100) (Developmental Studies Hybridoma Bank (DSHB)), mouse anti-Myc (9E10; 1∶50) (DSHB); rabbit anti-EGFP (1∶2000) (Abcam); rat anti-CD8a (1∶100) (Invitrogen); rat anti-Cut (1∶500); DyLight 488 AffiniPure Goat anti-HRP. Donkey anti-rat, anti-rabbit, and anti-mouse secondary antibodies were used at 1∶200–1∶300 (Jackson Immunoresearch). IHC slides were then imaged on a Nikon C1 Plus confocal microscope.

### Confocal Microscopy and Live Imaging

For live image analyses, third instar larvae were placed on a microscope slide, immersed in a few drops of 1∶5 (v/v) diethyl ether to halocarbon oil and covered with a 22×50 cm glass coverslip. Neurons expressing GFP were visualized using a Nikon C1 plus confocal microscope using the Nikon EZ-C1 software. Images were collected as z stacks at a step size of 1.5 µm and 1024×1024 pixel resolution. Z-stacks were then rendered into a maximum projection and resultant images were processed for quantitative neuronal reconstruction analyses.

### Neuronal Reconstruction, Morphometric Data Analyses and Statistics

Representative neurons from loss of function and gain of function analyses were selected for quantitation based on image quality and the absence of disrupted dendritic branches. Quantification of dendritic arbor complexity from representative neurons was performed by collecting z-series images acquired on a Nikon C1 Plus confocal microscope using a 20× (0.75 N.A.) or 40× (1.3 N.A.) objective, projected into a 2D image and imported into the Neuromantic software package for generation of neuronal reconstructions (.swc files) (http://www.reading.ac.uk/neuromantic/). Reconstruction files (.swc) were then input into the L-Measure software package [53] (http://cng.gmu.edu:8080/Lm/) and assigned parameters including total dendritic length, number of terminals, and dendritic branch order. Based upon these data, the average length per dendritic branch was calculated. For class III analyses, 100×100 micron boxes were drawn in areas proximal and distal to the cell body and the average number of dendritic terminals quantified in wild-type, *trio-RNAi* and Trio gain-of-function images. In the case of class IV ddaC neurons, the percentage field coverage is calculated by first drawing a box around the image which covers the maximum field over which class IV dendrites could extend. This box extends along the dorsal-ventral axis from the dorsal midline to the point where ddaC neuron dendrites tile with the lateral class IV v'ada neuron, then along the anterior-posterior axis, the box extends from anterior to posterior boundaries of an individual larval hemisegment. The area of this box is then calculated as the maximum field that class IV ddaC dendrites could cover and this represents the Expected_Area_. The actual field coverage is determined by calculated the area covered by the class IV dendrites using the polygon method as previously described [40] and this represents the Actual_Area_. Finally, the percentage field coverage by class IV dendrites was determined as follows: (Expected_Area_−Actual_Area_/Expected_Area_) (x) 100. In addition, total dendritic area for class I neurons was measured using the polygon method. Statistical analyses were performed in SigmaPlot (Systat Software) using Student's *t*-tests or Mann-Whitney rank sum tests. Dendritic branch order analyses were performed by computing branch order frequency distributions from whole neuron reconstructions via the branch order function of L-measure software. SigmaPlot was further used for data plotting and generating the fitted curves for the dendritic branch order analyses using the 5 Parameter Modified Gaussian Peak distribution equation for class I vpda neurons and the 4 Parameter Weibull Peak distribution equation for class IV ddaC neurons.

The relative expression levels of the Trio-GEF1-myc and Trio-GEF2-myc transgenes were quantified essentially as previously describe [52]. Briefly, confocal z-stack images were collected using identical settings for laser power and gain together with an equivalent step size between experimental samples. Z-sections were projected into 2D images and imported in Photoshop (Adobe) for measurements of integrated pixel density by area. The outline of each class IV ddaC cell body was traced based upon the HRP signal and then the fluorescence intensity for the Myc channel was determined and normalized to Cut expression levels in class IV neurons in order to control for any staining variation between samples. The normalized data was then used to examine the relative fluorescence intensities values between the GEF1-myc and GEF2-myc transgenes. A similar method was likewise used to quantify the relative fluorescence intensity levels for Cut-induced expression of Trio. In this case, Trio expression levels in class I neurons was quantified following Cut ectopic overexpression in these neurons and was normalized to normal Trio expression in class III neurons in which the *GAL4^221^,UAS-mCD8::GFP* driving *UAS-cut* is not expressed.

## Supporting Information

Figure S1 **Trio overexpression disrupts average dendritic branch length and field coverage in class IV da neurons.** (**A**) The average length per dendritic branch is not significantly altered with Trio overexpression, however Trio-GEF1 overexpression leads to a reduction, whereas Trio-GEF2 overexpression leads to an increase. (**B**) The percentage of dendritic field coverage is significantly reduced with Trio (81%), Trio-GEF1 (70%), and Trio-GEF2 (79%) overexpression as compared to controls (95%) reflecting defects in branching and growth. The total *n* value for each neuron and genotype quantified is reported on the bar graph. Statistically significant *p* values are reported on the graphs as follows (* = *p*<0.05; *** = *p*<0.001; n.s. = not significant). Genotypes: **WT**: *GAL4^477^,UAS-mCD8::GFP*/+. **TRIO**: *UAS-trio/+*;*GAL4^477^,UAS-mCD8::GFP/+*. **GEF1**: *UAS-trio-GEF1-myc/GAL4^477^,UAS-mCD8::GFP.* **GEF2**: *GAL4^477^,UAS-mCD8::GFP/+;UAS-trio-GEF2-myc/+*.
(TIF)

Figure S2 **Differential expression levels of the Trio-GEF1-myc and Trio-GEF2-myc transgenes.** (**A–H**) Representative confocal images of third instar larval class IV ddaC neurons expressing the *UAS-trio-GEF1-myc* transgene (**A–D**) or *UAS-trio-GEF2-myc* transgene (**E–H**) driven by *GAL4^477^,UAS-mCD8::GFP*. Larval filets were triple staining with HRP to visualize PNS neurons, anti-Myc to label the GEF1 vs. GEF2 expression levels, and Cut in order to normalize the Myc expression levels for potential variation between samples. (**I**) Quantitative analyses of relative fluorescence intensity values, normalized to Cut, reveal a mild, but significantly high level of Myc expression in the Trio-GEF1 transgene as compared to Trio-GEF2. The total *n* value for each neuron and genotype quantified is reported on the bar graph. Statistically significant *p* values are reported on the graphs as follows (* = *p*<0.05).
(TIF)

Figure S3 **Co-overexpression of GEF1 and GEF2 shifts branch order distribution relative to full length Trio overexpression.** (**A,B**) Representative live confocal images of third instar larval class IV ddaC neurons labelled with *ppkGAL4,UAS-mCD8::GFP* (n = 8). Size bar represents 50 microns. As compared to full length Trio overexpression (**A**), co-overexpression of Trio-GEF1 and Trio-GEF2 results in a qualitative change in branch order distribution. (**C**) Morphometric reconstruction analyses reveal a distal shift towards an increased percentage of higher order branches in GEF1-GEF2 co-overexpression relative to full length Trio overexpression consistent with the qualitative phenotypic data. Genotypes: **TRIO**: *UAS-trio/+;+;ppkGAL4,UASmCD8::GFP/+*. **GEF1+GEF2**: *UAS-trio-GEF1-myc/+;ppkGAL4,UASmCD8::GFP/UAS-trio-GEF2-myc*.
(TIF)

Figure S4 **Cut overexpression upregulates Trio in da neurons.** Quantitative analyses of relative fluorescence intensities for Trio were performed in class I da neurons in the presence or absence of Cut overexpression. Trio fluorescence intensity values in the control and experimental samples were normalized against normal Trio fluorescence intensity levels in adjacent class III da neurons which do not express the *GAL4^221^,UAS-mCD8::GFP* reporter. These analyses revealed an approximate 10% increase in Trio fluorescence intensity in class I neurons ectopically overexpressing Cut relative to controls in the absence of Cut overexpression. The total *n* value for genotype quantified is reported on the bar graph. Statistically significant *p* values are reported on the graphs as follows (*** = *p*<0.001).
(TIF)

Figure S5 **Co-expression of Cut and Trio reveals synergistic effects on dendrite development.** (**A–D**) Representative live confocal images of third instar larval class I vpda neurons labeled with *GAL4^221^,UAS-mCD8::GFP* (n = 10). Size bars represent 100 microns. (**A**) Ectopic expression of Cut in class I neurons leads *de novo* dendritic branching and promotes dendritic extension resulting in a significant increase in complexity and length. (**B**) Co-expression of Cut and full length Trio reveals a moderate phenotypic increase in branching. (**C**) Co-expression of Cut and Trio-GEF1 results in a strong phenotypic increase in dendritic branching complexity. (**D**) Co-expression of Cut and Trio-GEF2 primarily results in increased dendritic extension. (**E**) Cut synergistically acts with full length Trio and Trio-GEF1 in promoting dendritic branching complexity, whereas no significant effect is observed with Trio-GEF2. (**F**) Cut synergistically acts with Trio, Trio-GEF1, and Trio-GEF2 to increase total dendritic length through increased overall branching and/or dendritic extension. The total *n* value for each neuron and genotype quantified is reported on the bar graph. Statistically significant *p* values are reported on the graphs as follows (* = *p*<0.05; ** = *p*<0.01; *** = *p*<0.001; n.s. = not significant). Genotypes: **CUT**: *UAS-cut/+;GAL4^221^,UASmCD8::GFP/+*. **CUT+TRIO**: *UAS-trio/+;UAS-cut/+;GAL4^221^,UASmCD8::GFP/+*. **CUT+GEF1**: *UAS-trio-GEF1-myc/UAS-cut;GAL4^221^,UASmCD8::GFP/+*. **CUT+GEF2**: *UAS-cut/+;UAS-trio-GEF2-myc/GAL4^221^,UASmCD8::GFP/+*.
(TIF)
