# Supplementary figures and images for: The RhoGEF Trio Functions in Sculpting Class Specific Dendrite Morphogenesis in Drosophila Sensory Neurons
Source: PLoS One. 2012 Mar 19;7(3):e33634. doi: 10.1371/journal.pone.0033634 (PMC3307743; doi:10.1371/journal.pone.0033634)

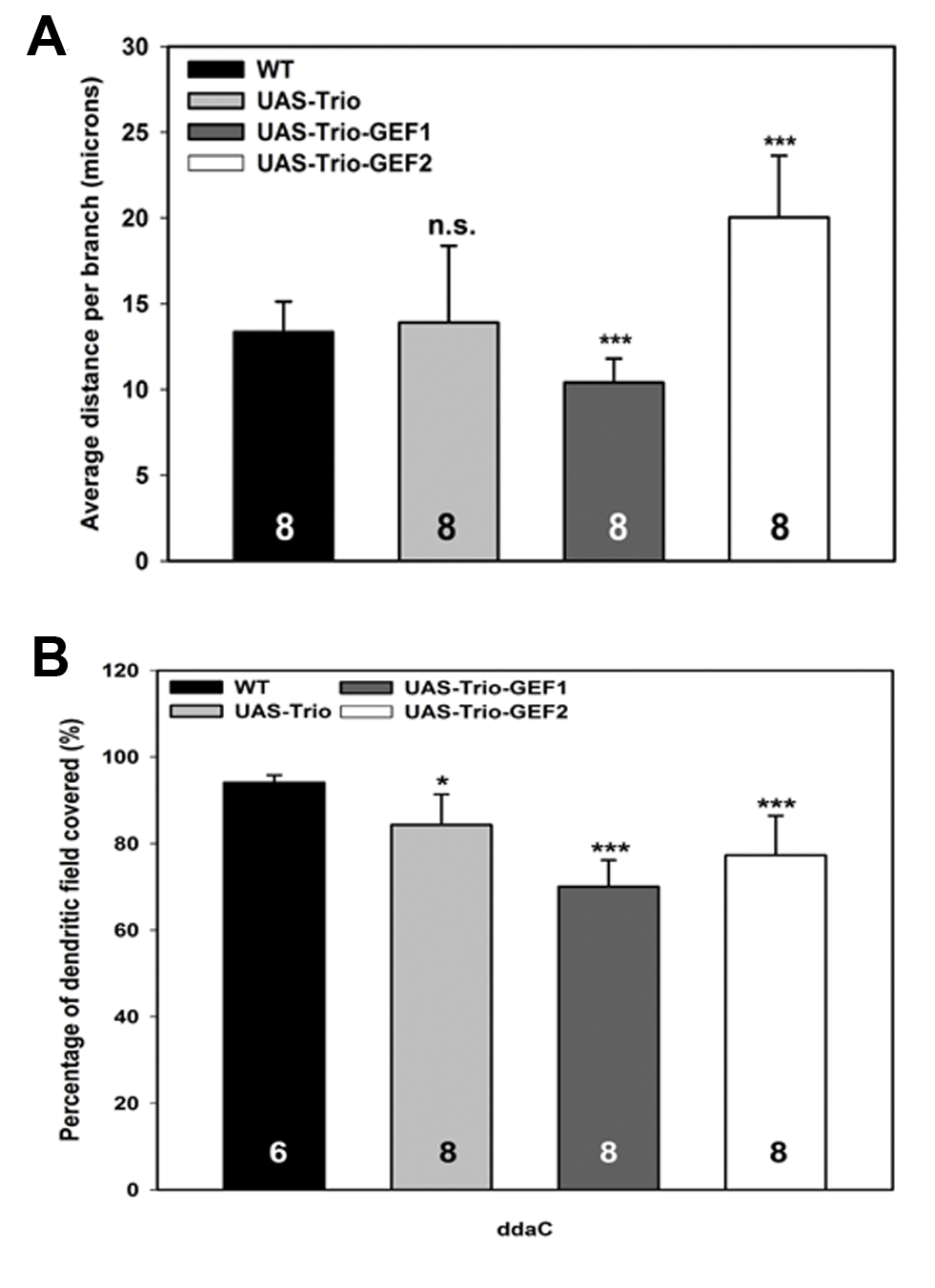

Supplement: Figure S1 — Trio overexpression disrupts average dendritic branch length and field coverage in class IV da neurons. (A) The average length per dendritic branch is not significantly altered with Trio overexpression, however Trio-GEF1 overexpression leads to a reduction, whereas Trio-GEF2 overexpression leads to an increase. (B) The percentage of dendritic field coverage is significantly reduced with Trio (81%), Trio-GEF1 (70%), and Trio-GEF2 (79%) overexpression as compared to controls (95%) reflecting defects in branching and growth. The total n value for each neuron and genotype quantified is reported on the bar graph. Statistically significant p values are reported on the graphs as follows (* = p<0.05; *** = p<0.001; n.s. = not significant). Genotypes: WT: GAL4477,UAS-mCD8::GFP/+. TRIO: UAS-trio/+;GAL4477,UAS-mCD8::GFP/+. GEF1: UAS-trio-GEF1-myc/GAL4477,UAS-mCD8::GFP. GEF2: GAL4477,UAS-mCD8::GFP/+;UAS-trio-GEF2-myc/+. (TIF) [file pone.0033634.s001.tif]

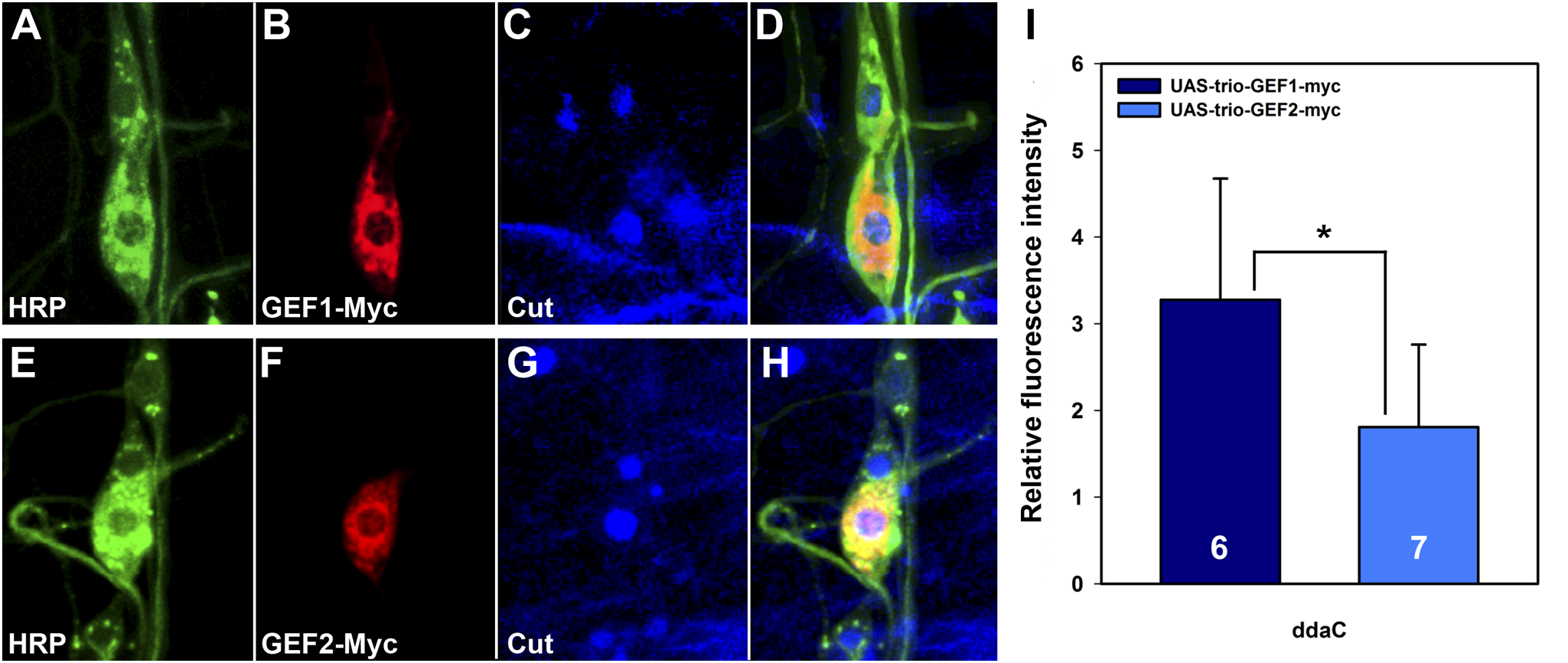

Supplement: Figure S2 — Differential expression levels of the Trio-GEF1-myc and Trio-GEF2-myc transgenes. (A–H) Representative confocal images of third instar larval class IV ddaC neurons expressing the UAS-trio-GEF1-myc transgene (A–D) or UAS-trio-GEF2-myc transgene (E–H) driven by GAL4477,UAS-mCD8::GFP. Larval filets were triple staining with HRP to visualize PNS neurons, anti-Myc to label the GEF1 vs. GEF2 expression levels, and Cut in order to normalize the Myc expression levels for potential variation between samples. (I) Quantitative analyses of relative fluorescence intensity values, normalized to Cut, reveal a mild, but significantly high level of Myc expression in the Trio-GEF1 transgene as compared to Trio-GEF2. The total n value for each neuron and genotype quantified is reported on the bar graph. Statistically significant p values are reported on the graphs as follows (* = p<0.05). (TIF) [file pone.0033634.s002.tif]

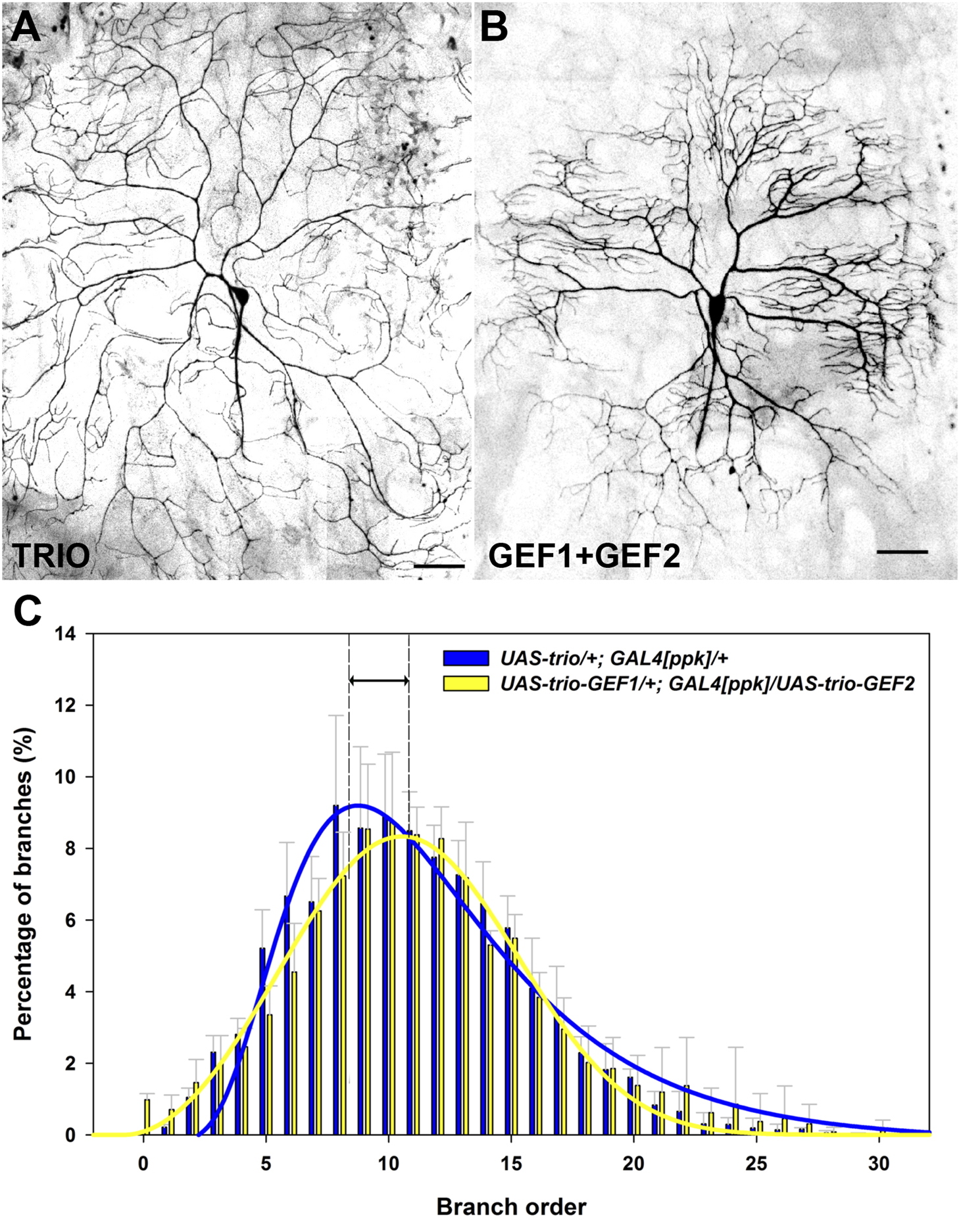

Supplement: Figure S3 — Co-overexpression of GEF1 and GEF2 shifts branch order distribution relative to full length Trio overexpression. (A,B) Representative live confocal images of third instar larval class IV ddaC neurons labelled with ppkGAL4,UAS-mCD8::GFP (n = 8). Size bar represents 50 microns. As compared to full length Trio overexpression (A), co-overexpression of Trio-GEF1 and Trio-GEF2 results in a qualitative change in branch order distribution. (C) Morphometric reconstruction analyses reveal a distal shift towards an increased percentage of higher order branches in GEF1-GEF2 co-overexpression relative to full length Trio overexpression consistent with the qualitative phenotypic data. Genotypes: TRIO: UAS-trio/+;+;ppkGAL4,UASmCD8::GFP/+. GEF1+GEF2: UAS-trio-GEF1-myc/+;ppkGAL4,UASmCD8::GFP/UAS-trio-GEF2-myc. (TIF) [file pone.0033634.s003.tif]

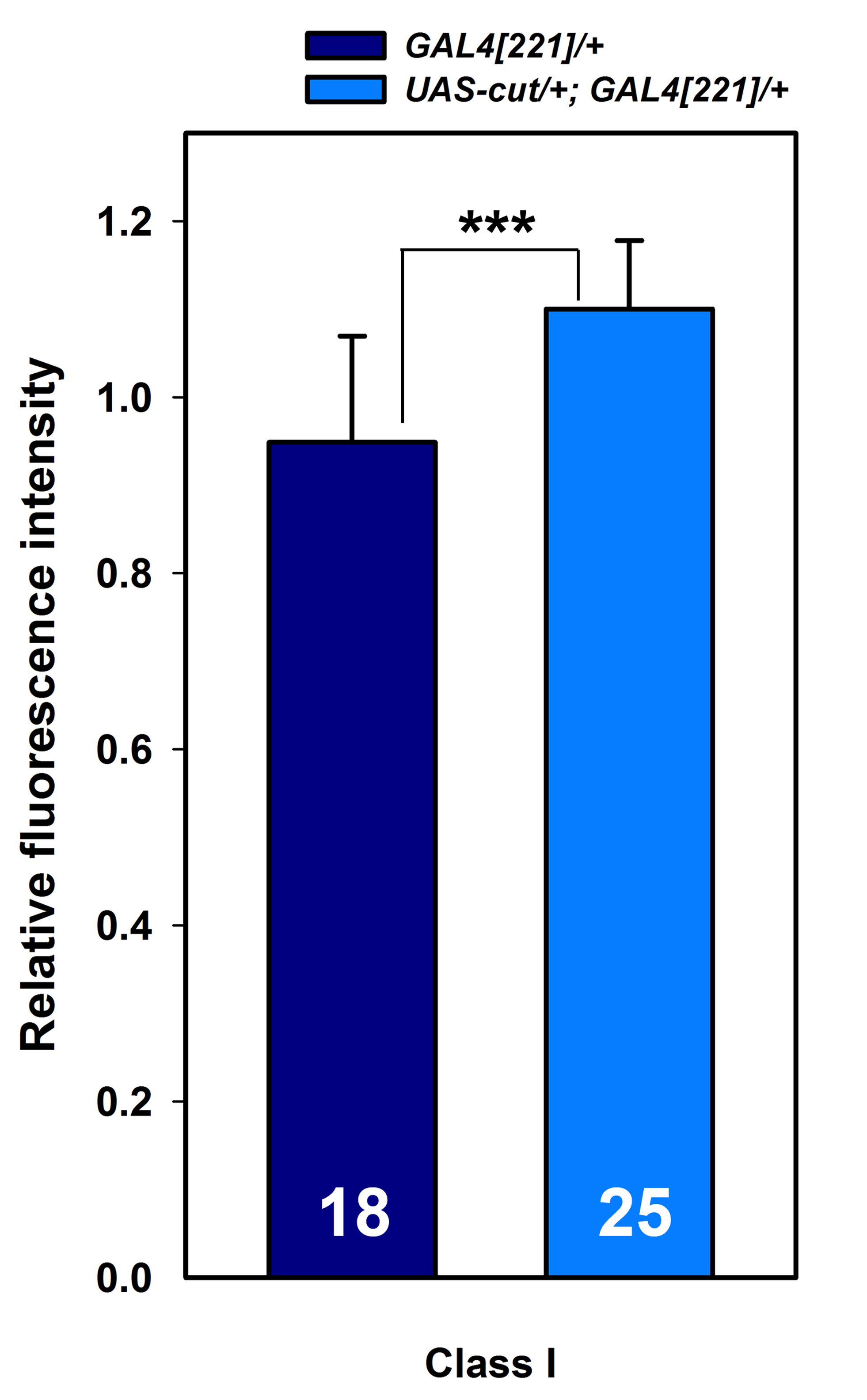

Supplement: Figure S4 — Cut overexpression upregulates Trio in da neurons. Quantitative analyses of relative fluorescence intensities for Trio were performed in class I da neurons in the presence or absence of Cut overexpression. Trio fluorescence intensity values in the control and experimental samples were normalized against normal Trio fluorescence intensity levels in adjacent class III da neurons which do not express the GAL4221,UAS-mCD8::GFP reporter. These analyses revealed an approximate 10% increase in Trio fluorescence intensity in class I neurons ectopically overexpressing Cut relative to controls in the absence of Cut overexpression. The total n value for genotype quantified is reported on the bar graph. Statistically significant p values are reported on the graphs as follows (*** = p<0.001). (TIF) [file pone.0033634.s004.tif]

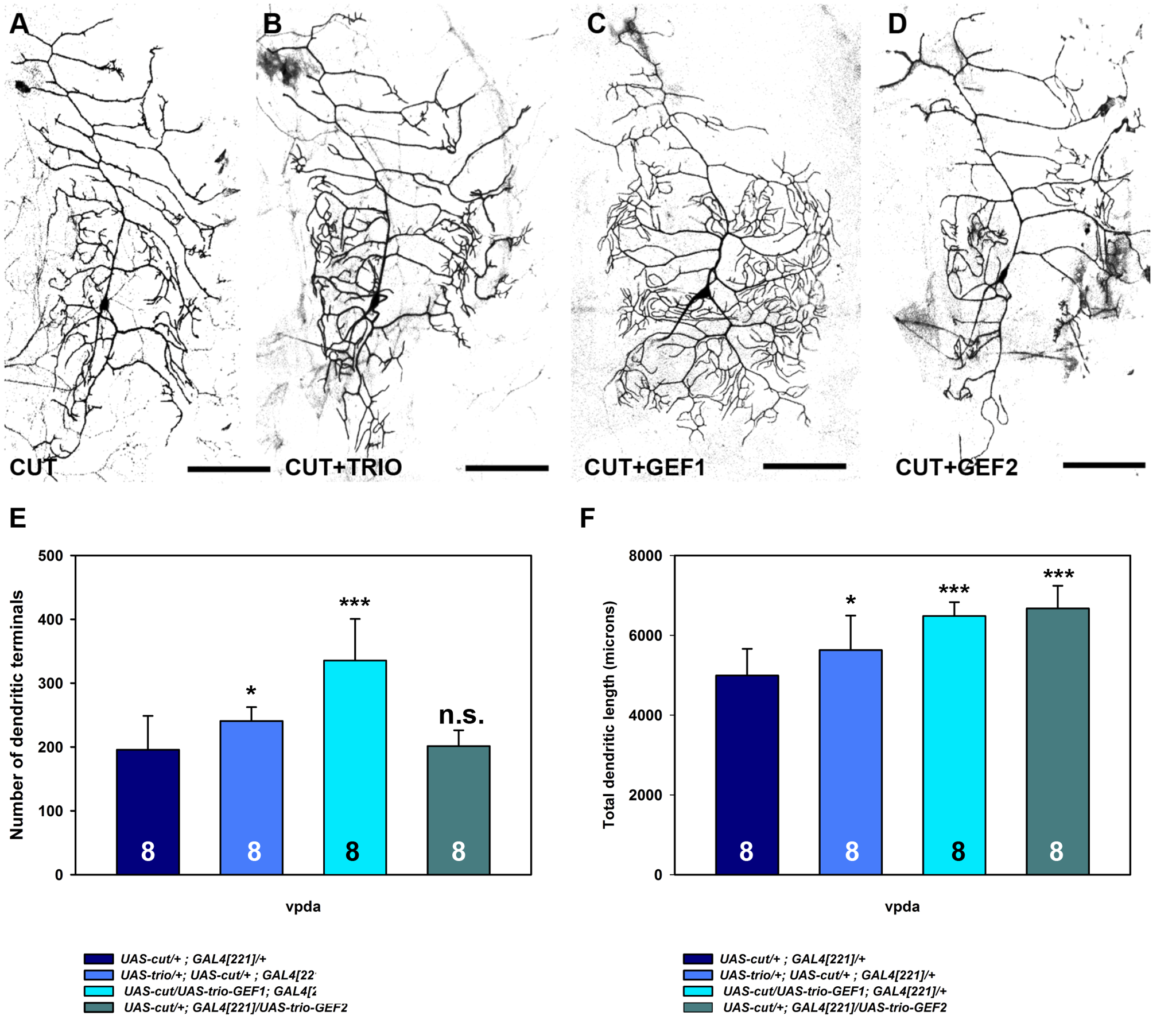

Supplement: Figure S5 — Co-expression of Cut and Trio reveals synergistic effects on dendrite development. (A–D) Representative live confocal images of third instar larval class I vpda neurons labeled with GAL4221,UAS-mCD8::GFP (n = 10). Size bars represent 100 microns. (A) Ectopic expression of Cut in class I neurons leads de novo dendritic branching and promotes dendritic extension resulting in a significant increase in complexity and length. (B) Co-expression of Cut and full length Trio reveals a moderate phenotypic increase in branching. (C) Co-expression of Cut and Trio-GEF1 results in a strong phenotypic increase in dendritic branching complexity. (D) Co-expression of Cut and Trio-GEF2 primarily results in increased dendritic extension. (E) Cut synergistically acts with full length Trio and Trio-GEF1 in promoting dendritic branching complexity, whereas no significant effect is observed with Trio-GEF2. (F) Cut synergistically acts with Trio, Trio-GEF1, and Trio-GEF2 to increase total dendritic length through increased overall branching and/or dendritic extension. The total n value for each neuron and genotype quantified is reported on the bar graph. Statistically significant p values are reported on the graphs as follows (* = p<0.05; ** = p<0.01; *** = p<0.001; n.s. = not significant). Genotypes: CUT: UAS-cut/+;GAL4221,UASmCD8::GFP/+. CUT+TRIO: UAS-trio/+;UAS-cut/+;GAL4221,UASmCD8::GFP/+. CUT+GEF1: UAS-trio-GEF1-myc/UAS-cut;GAL4221,UASmCD8::GFP/+. CUT+GEF2: UAS-cut/+;UAS-trio-GEF2-myc/GAL4221,UASmCD8::GFP/+. (TIF) [file pone.0033634.s005.tif]
